# Impaired Increase of Plasma Abscisic Acid in Response to Oral Glucose Load in Type 2 Diabetes and in Gestational Diabetes

**DOI:** 10.1371/journal.pone.0115992

**Published:** 2015-02-27

**Authors:** Pietro Ameri, Santina Bruzzone, Elena Mannino, Giovanna Sociali, Gabriella Andraghetti, Annalisa Salis, Monica Laura Ponta, Lucia Briatore, Giovanni F. Adami, Antonella Ferraiolo, Pier Luigi Venturini, Davide Maggi, Renzo Cordera, Giovanni Murialdo, Elena Zocchi

**Affiliations:** 1 Department of Internal Medicine, University of Genova, Viale Benedetto XV/6, 16132 Genova, Italy; 2 Department of Experimental Medicine, Section of Biochemistry and CEBR, University of Genova, Viale Benedetto XV/1, 16132 Genova, Italy; 3 Department of surgical sciences and integrated diagnostic, University of Genova, Largo R. Benzi 8, 16132 Genova, Italy; 4 IRCSS San Martino IST, Largo R. Benzi 10, 16132 Genova, Italy; 5 Department of Neurosciences, Rehabilitation, Ophthalmology, Genetics and Mother and Child Sciences, University of Genova, Largo P. Daneo, 3, 16132 Genova, Italy; Consiglio Nazionale delle Ricerche, ITALY

## Abstract

The plant hormone abscisic acid (ABA) is present and active in humans, regulating glucose homeostasis. In normal glucose tolerant (NGT) human subjects, plasma ABA (ABAp) increases 5-fold after an oral glucose load. The aim of this study was to assess the effect of an oral glucose load on ABAp in type 2 diabetes (T2D) subjects. We chose two sub-groups of patients who underwent an oral glucose load for diagnostic purposes: i) 9 treatment-naive T2D subjects, and ii) 9 pregnant women with gestational diabetes (GDM), who underwent the glucose load before and 8–12 weeks after childbirth. Each group was compared with matched NGT controls. The increase of ABAp in response to glucose was found to be abrogated in T2D patients compared to NGT controls. A similar result was observed in the women with GDM compared to pregnant NGT controls; 8–12 weeks after childbirth, however, fasting ABAp and ABAp response to glucose were restored to normal in the GDM subjects, along with glucose tolerance. We also retrospectively compared fasting ABAp before and after bilio-pancreatic diversion (BPD) in obese, but not diabetic subjects, and in obese T2D patients, in which BPD resulted in the resolution of diabetes. Compared to pre-BPD values, basal ABAp significantly increased 1 month after BPD in T2D as well as in NGT subjects, in parallel with a reduction of fasting plasma glucose. These results indicate an impaired hyperglycemia-induced ABAp increase in T2D and in GDM and suggest a beneficial effect of elevated ABAp on glycemic control.

## Introduction

The phytohormone abscisic acid (ABA) lies at the interface between abiotic stress and metabolic signaling in plants, regulating vital functions [1]. Interestingly, ABA was found to play a conserved role as a “stress hormone” also in lower Metazoa, regulating animal responses to temperature and light [2, 3], and in mammals, regulating the activation of innate immune cells [4, 5] and glucose homeostasis [6–8].

Mammalian pancreatic β-cells release ABA in response to glucose and ABA in turn potentiates glucose-dependent and stimulates glucose-independent insulin secretion, suggesting the existence of an autocrine/paracrine feed-forward loop [6]. ABA also stimulates GLUT4-mediated glucose uptake by adipocytes and myoblasts [7]. Plasma concentration of ABA (ABAp) increases in healthy subjects after an oral glucose load and ABAp is higher when glucose is administered orally than intravenously [7], indicating a stimulatory effect of incretins on ABA release, similar to the one on insulin. Indeed, glucagon-like peptide 1 (GLP-1) enhances ABA secretion from glucose-stimulated pancreatic β-cells *in vitro* [7]. ABA role in glucose homeostasis prompts the hypothesis that diabetes mellitus is associated with an impaired ABA response. Pointedly, in the *db/db* mouse model of obesity-induced type 2 diabetes (T2D), supplementation of a high-fat diet with ABA results in improved glucose tolerance [8].

The aim of this study was to prospectively assess the effect of an oral glucose load on ABAp in T2D subjects and in pregnant women with gestational diabetes (GDM), each group being compared with appropriate normal glucose tolerant (NGT) controls. In addition, we retrospectively compared fasting ABAp before and after bilio-pancreatic diversion (BPD) in obese, but not diabetic subjects, and in obese T2D patients. In the latter group, BPD resulted in the resolution of diabetes, as generally occurs [9, 10], thus providing another “on-off” model of the disease, in addition to GDM, in which to investigate the behaviour of ABAp.

## Methods

### Study subjects and sampling procedures

The study of ABAp in T2D included a total of 50 subjects. Eleven of them had impaired fasting glucose levels and underwent a standard oral glucose tolerance test (OGTT) for diagnostic purposes after overnight fasting. Plasma samples were collected immediately before (time zero) and 15, 30, 60, 90, and 120 minutes after ingestion of 75 g glucose. Two-ml plasma samples for each time point were immediately frozen in the presence of 4 vol distilled methanol for subsequent ABAp measurement. Glucose, insulin, and ABAp were then measured in 9 out of the 11 subjects, who were diagnosed with T2D on the basis of the result of the OGTT. The same OGTT and sampling procedure was applied to 7 healthy volunteers of comparable age and body mass index (BMI), who served as controls. Fasting plasma glucose (FPG) and ABAp were also measured in 12 treatment-naïve T2D patients and in 20 age-and BMI-matched healthy volunteers. Therefore, fasting ABAp and FPG values were assessed in a total of 21 T2D patients (9 undergoing an OGTT) and 27 controls (7 undergoing an OGTT). The diabetic patients did not have any other acute or chronic disease, and followed an unrestricted diet, as the normal subjects.

The study of ABAp in GDM included a total of 16 women. All underwent a standard screening for gestational diabetes between the 24^th^ and the 28^th^ week of gestation and agreed to repeat the test 8–12 weeks after childbirth. After overnight fasting, plasma samples for glucose and ABAp and insulin determinations were collected immediately before (time zero) and 60 and 120 minutes after ingestion of 75 g of glucose. Out of the 16 women studied, 9 were diagnosed with GDM, based on the finding of plasma glucose values ≥180 mg/dL or ≥153 mg/dL 1 or 2 hours after the glucose load, respectively [11]. None of the women reported a change in her dietary habits after childbirth.

In the retrospective part of the study, ABAp was measured in frozen plasma samples from 20 severely obese patients who had undergone BPD within a clinical study and had their blood withdrawn 1 week before and 1 month after surgery [12]. Nine subjects had T2D before BPD, while the others had normal FPG levels and served as controls. FPG and insulin concentrations had been determined at the time of sample collection.

The present study was approved by the Local Ethics Committee (IRCCS AOU San Martino-IST, Genoa, Italy) and was conducted according to the Declaration of Helsinki principles. Written informed consent was given by every participant.

### Measurements

All measurements were made in duplicate in the same batch. Plasma glucose concentration was measured by an enzymatic method (Randox Laboratories, Crumlin, UK); plasma insulin concentration was determined by a sandwich immunoradiometric assay (Immunotech, Prague, Czech Republic), as described in [7]. Homeostasis model assessment of insulin resistance (HOMA-IR) was calculated as [fasting insulin (pmol/L) × fasting glucose (mmol/L)]/135. In the prospective study involving T2D patients, fasting ABAp was determined by both high-performance liquid chromatography-mass spectrometry (HPLC-MS) [7] and enzyme-linked immunoassay (ELISA; Agdia Biofords, Evry CEDEX, France). A good agreement between the results obtained with the two techniques was observed. Thus, ABAp at the different time points of the OGTT was determined by ELISA only. In the plasma samples from the pregnant women, ABAp was measured by ELISA only. In the retrospective study, plasma samples collected at the time of the bariatric surgery and kept at -20°C were extracted in 4 vol distilled methanol and ABAp was determined by ELISA [7].

The area-under-the-curve (AUC) values of plasma ABA, glucose and insulin were calculated with the trapezoidal rule, from the concentrations measured at the time points indicated in the legends to Table 1 and Table 2.

**Table 1:** Impaired increase of the ABAp during OGTT in T2D patients.

	NGT	T2D	*p*
***N***	7	9	
**Age (years)**	58.1±6.2	60.2±10.2	0.642
**BMI (kg/m** ^**2**^)	30.2±6.1	27.7±4.4	0.342
**FPG (mg/dL)**	95.4±12.6	145.8±39.6	**0.007**
**Fasting ABAp**	0.97±0.36	1.75±1.02	**0.040**
**(nM)**	0.99 (0.38–1.54)	1.61 (0.35–3.42)	
ABA AUC (nmol/L*min)	121.7±33.9	68.3±44.3	**0.019**
Glucose AUC (mg/dL*min)	18550±4422	30694±6129	**<0.001**
Insulin AUC (mU/L*min)	6998±910	8192±2724	0.155

Study participants underwent an OGTT after overnight fasting. Plasma ABA, glucose and insulin concentrations were measured immediately before and 15, 30, 60, 90 and 120 min after the ingestion of 75 g of glucose. AUCs were calculated from the absolute values for glycemia and insulinemia, or from the values relative to time zero for ABAp. Data are presented as mean±SD and were compared by unpaired t-test (significant p values in bold). For ABAp, median value and range are also shown.

**Table 2:** Higher fasting ABAp in T2D patients compared to NGT controls.

	NGT	T2D	*p*
***N***	27	21	
**BMI (kg/m** ^**2**^)	26.3±6.3	29.9±7.7	0.197
**FPG (mg/dL)**	84.6±32.4	140.4±57.6	**0.003**
**Fasting ABAp (nM)**	0.74±0.45	1.68±1.44	**0.003**
	0.66 (0.13–1.72)	1.15 (0.19–4.77)	**0.013**§

Fasting plasma glucose (FPG) and ABA levels were determined in two groups of age- and sex (male)-matched subjects which included the participants to the OGTT shown in Table 1. Data are presented as mean ± SD or median (range) and are compared by t-test or Mann-Whitney test (§), respectively (significant p values in bold). For fasting ABAp, both mean and median values are shown, with the respective statistical comparison. ABAp was measured by HPLC-MS.

### Statistical analysis

Continuous variables are presented as mean±SD or median (interquartile range), depending on the distribution. Comparisons were drawn by chi-square test, unpaired or paired t-test, or Mann-Whitney test, as appropriate. The relationship between the AUC of glucose and of ABAp was studied by means of the Pearson correlation test. Statistical significance was set at *p*<0.05.

## Results

### Impaired increase of plasma ABA after glucose load in T2D subjects

**Fig 1:**
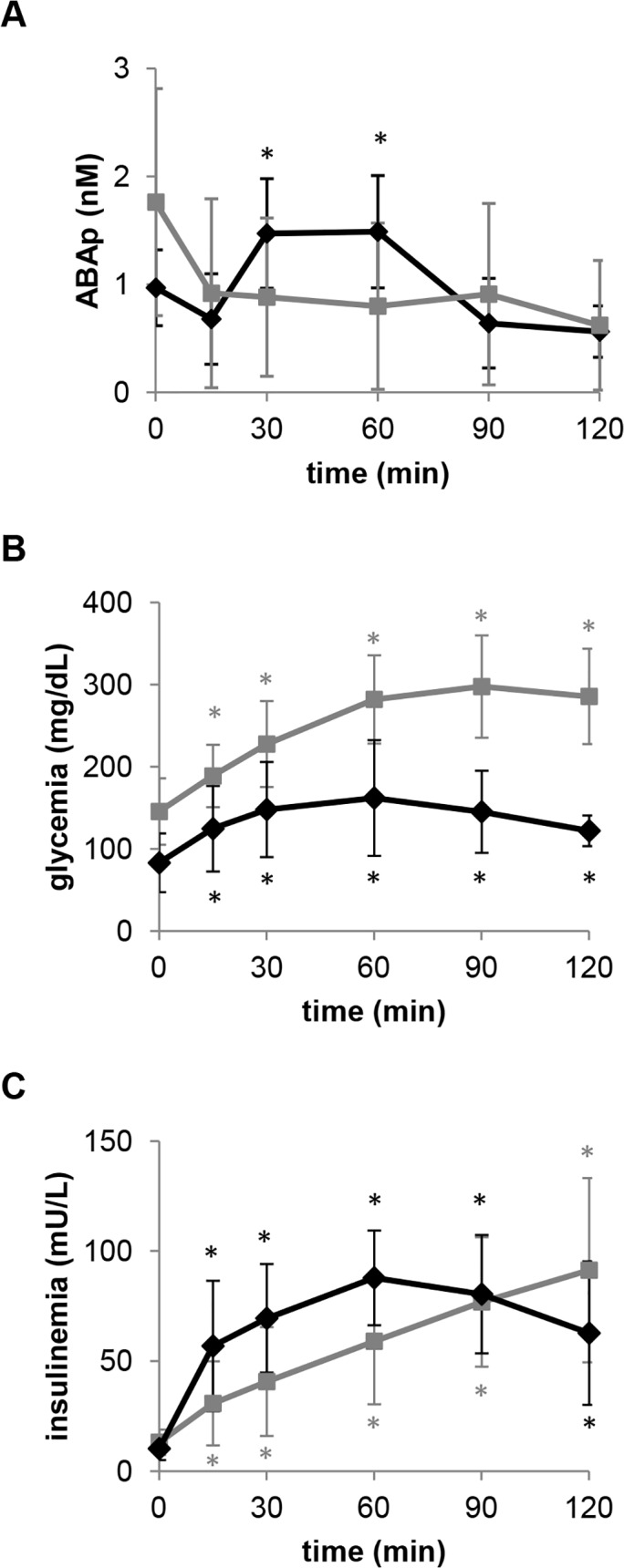
ABAp increases after an oral glucose load in healthy subjects, but not in T2D patients. After overnight fasting, a pre-test blood sample was taken from 7 healthy subjects and from 9 T2D patients, all of whom subsequently underwent a standard OGTT. The values of plasma ABA (A), glucose (B) and insulin (C) shown are the mean ± SD from the healthy controls (black rhombi) and from the T2D subjects (grey squares). * p<0.05 relative to time zero values.

Consistent with the experimental evidence supporting a role of ABA in promoting glucose disposal, ABAp increases following an oral glucose load in healthy individuals [7]. To determine whether the ABA response to oral glucose was modified in T2D, plasma samples from 11 patients with impaired fasting glycemia, who underwent an OGTT for diagnostic purposes, were collected immediately before and 15, 30, 60, 90, and 120 minutes after intake of glucose; in 9 out of these 11 subjects, T2D was confirmed by the test. Seven volunteers of comparable age and BMI and with normal glucose tolerance served as controls. ABAp increased in response to oral glucose in all healthy subjects, but not in the diabetic subjects. The curves of glycemia, insulinemia and ABAp following glucose ingestion in T2D subjects and controls are shown in Fig. 1. As a result of the failure of ABAp to increase in the T2D patients, the AUC of ABAp during the OGTT was significantly lower in these subjects compared to NGT controls (Table 1). In the control group, there was a positive correlation between the AUC of ABAp and the one of glycemia, (*r* = 0.7767, *p* = 0.040). This positive correlation confirms our previous observation [7] and supports the conclusion that, under normal conditions, hyperglycemia is a main driver to ABA secretion. Interestingly, no significant correlation was found between the AUC of ABAp and glycemia in T2D patients (*r* = -0.4247, *p* = 0.255). Altogether, these results suggest that the mechanism by which high glucose induces an increase in ABAp is impaired in T2D.

**Fig 2:**
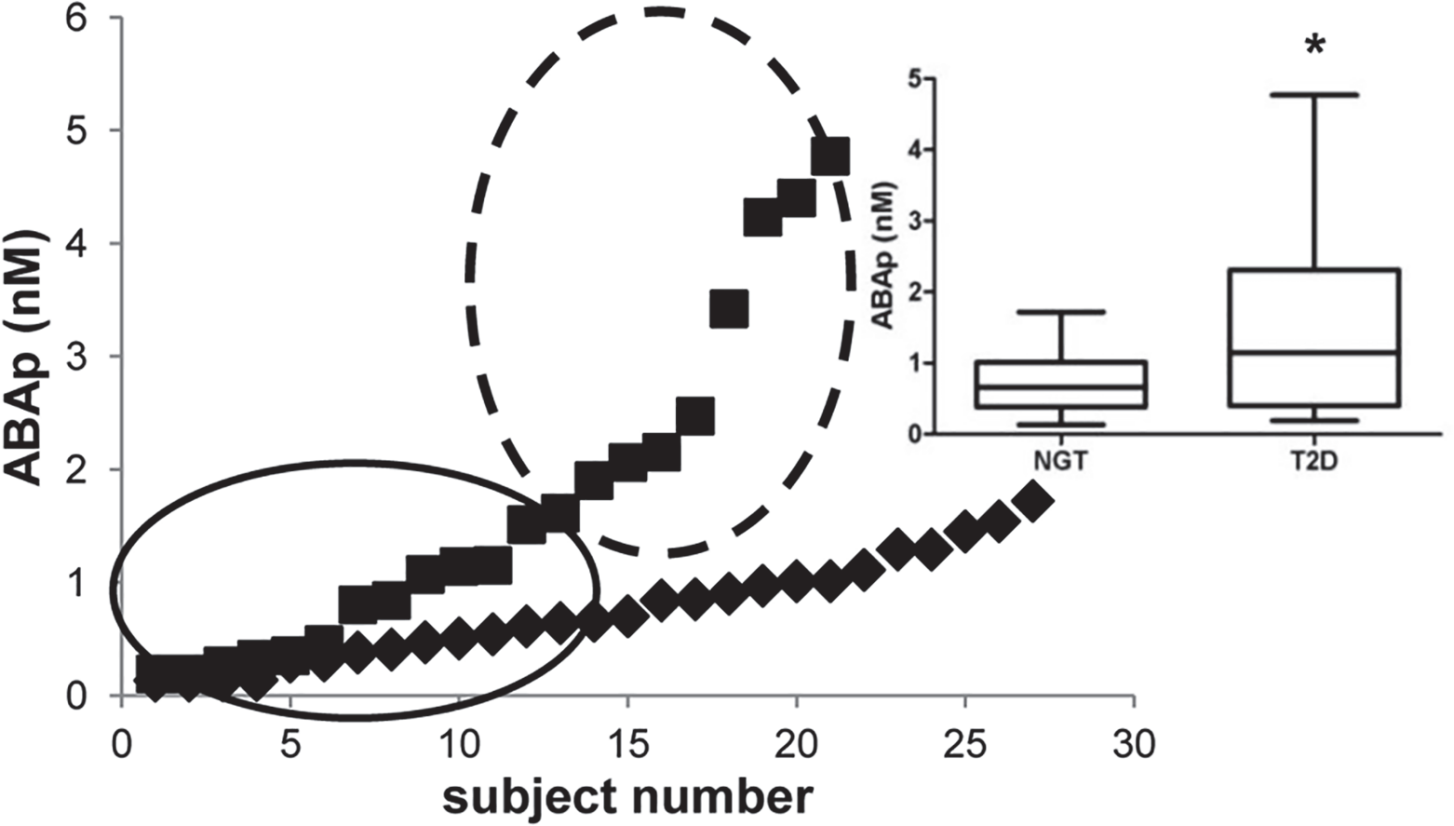
Fasting ABAp in NGT subjects and T2D patients. Fasting ABAp was determined by HPLC-MS in 21 male T2D patients (squares) and in 27 sex-, age- and BMI-matched NGT subjects (rhombi). Results are ordered by increasing value. The circled areas indicate the possible existence of two sub-groups within the T2D patients, one with higher-than-normal ABAp levels and one with ABAp values similar to those of the NGT group. Inset: a box-and-whisker plot drawn from the same data sets. * p = 0.013

We also compared fasting ABAp and FPG between a group of 21 treatment-naïve patients with T2D (including the 9 subjects who underwent the OGTT) and a group of 27 age- and BMI-matched healthy volunteers with normal glucose tolerance. As expected, FPG was significantly higher in the diabetic subjects compared to the controls (Table 2). Interestingly, the distribution of ABAp values was normal in NGT subjects, but not in T2D patients. Fig. 2 shows the individual ABAp values from all subjects: the distribution of ABAp values in the T2D subjects suggests the existence of two sub-groups, one with higher-than-normal ABAp and the other one with ABAp similar to normal. No significant correlation between ABAp and age or BMI was observed within the T2D group. The median value and the range were 0.66 (0.13–1.72) nM and 1.15 (0.19–4.77) nM in the healthy and in the T2D subjects, respectively. Analysis of the data by the Mann-Whitney test indicated a statistically significant difference between ABAp in NGT and T2D subjects (*p* = 0.013; Table 2).

### Diminished increase of ABAp after oral glucose load in GDM is followed by postpartum restoration of the ABAp response to hyperglycemia and resolution of diabetes

**Fig 3:**
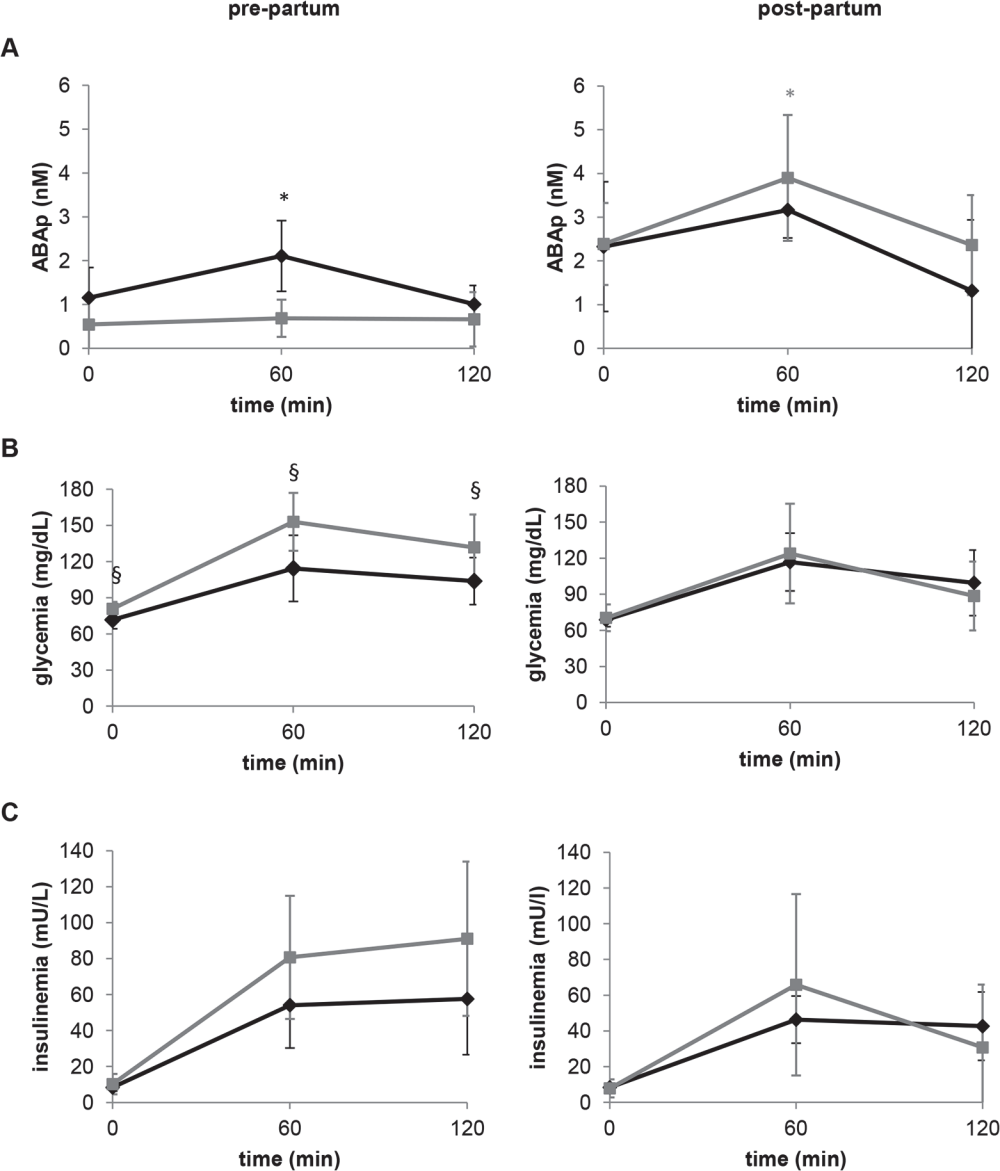
Pre-partum impairment and post-partum restoration of the ABAp increase after oral glucose load in GDM subjects. The values of plasma ABA (A), glucose (B) and insulin (C) shown are the mean ± SD from seven NGT (black rhombi) and from nine GDM subjects (grey squares), who underwent a standard OGTT at the 24^th^-28^th^ week (pre-partum) and again 2–3 months after childbirth (post-partum). Post-partum restoration of the ABAp increase during OGTT in the GDM subjects was accompanied by restoration of a normal glycemic profile. * p<0.05 compared to time zero values; ^§^ p<0.05 compared to NGT.

To investigate whether the ABAp response to oral glucose was also altered in GDM, plasma samples were collected during in-pregnancy and post-partum OGTTs in 16 women. Nine of them had GDM, which reverted to NGT after childbirth; the other subjects were already NGT during their pregnancy (Table 3). The curves of glycemia, insulinemia and ABAp following glucose ingestion in GDM and NGT women, pre- and post-partum, are shown in Fig. 3. Both the maximal ABAp increase and the ABA AUC after the oral glucose load were significantly lower in women with GDM compared to those with NGT during pregnancy. In the GDM group, the AUC of ABAp was significantly higher after childbirth compared to the in-pregnancy values. A similar trend was also observed in NGT subjects, although to a minor extent (Table 3). As a result, 8–12 weeks after childbirth the ABAp AUC was no longer significantly different between women diagnosed with GDM and controls. Concomitant with the restoration of the ABAp response to hyperglycemia, normalization of glucose tolerance was observed in all GDM subjects (Table 3). Interestingly, after childbirth, there was a significant positive correlation between the AUC of ABAp and the one of glycemia in both the GDM and the NGT group (*r* = 0.9222, *p =* 0.00031 and *r* = 0.7478, *p* = 0.020, respectively). This correlation was also observed in the control subjects of Table 1 (see first paragraph of the Results) and in a previous study in healthy subjects [7].

**Table 3:** Diminished increase of ABAp after oral glucose load in GDM and reversal to normal after childbirth.

		NGT			GDM		
*N*		7			9		*p*
BMI (kg/m^2^)		24.9±3.1			25.5±4.9		0.767
Age (years)		37.0±3.7			37.1±1.4		0.934
	*prepartum*		*postpartum*	*prepartum*		*postpartum*	*p*
FPG (mg/dL)	72.4±7.2	*p* = 0.117	67.9±9.3	80.7±5.8	*p* = **0.007**	70.3±11.1	**0.015** * 0.631#
Fasting ABAp (nM)	1.15±0.69	*p* = **0.011**	2.33±1.49	0.54±0.62	*p* = **0.0002**	2.39±0.98	**0.043** * 0.708#
ABA AUC (nmol/L*min)	191.2±78.3	*p* = 0.113	298.3±105.2	79.4±56.9	*p* = **0.011**	376.5±98.9	**0.033** * 0.113#
Glucose AUC (mg/dL*min)	12604±256	*p* = 0.686	12116±2182	15546±2066	*p* = **0.022**	12258±3084	**0.015** * 0.914#
Insulin AUC (mU/L*min)	5984±2942	*p* = 0.284	4911±1889	7465±3406	*p* = **0.015**	5438±3641	0.237* 0.894#

Plasma ABA, glucose and insulin concentrations were measured at 0, 60 and 120 min during a standard gestational diabetes screen at the 24^th^-28^th^ week (prepartum). The same test was repeated 8–12 weeks after childbirth (postpartum). ABAp was measured by ELISA.

*unpaired t test NGT prepartum vs GDM prepartum

#unpaired t test NGT postpartum vs GDM postpartum.

Interestingly, pre-partum fasting ABAp was significantly lower in GDM subjects (0.54±0.62 nM, n = 10) compared to NGT controls (1.15±0.69 nM, n = 12, *p* = 0.043). After childbirth, ABAp increased significantly in both NGT and GDM subjects, in the latter ones reaching control values (Table 3).

Altogether, these results indicate that the transient diabetic condition of GDM is associated with a similarly transient impairment of the hyperglycemia-induced increase of ABAp and that post-partum restoration of NGT is concomitant with a normalization of the ABAp response to hyperglycemia.

### Fasting ABAp increases after BPD

In the retrospective part of this study, we compared the fasting ABAp in morbidly obese diabetic and non-diabetic subjects, before and 1 month after BPD. This type of bariatric surgery very often results in the rapid (within 1 month) resolution of diabetes [10]. Before BPD, FPG, insulin, and HOMA-IR were significantly higher in T2D than in NGT obese patients, as expected (Table 4). Fasting ABAp was also higher in the first group than in the latter, in agreement with what observed in the prospective cohort of non-obese T2D patients (Table 2). One month after BPD, in parallel with a significant reduction of the BMI, FPG, HOMA-IR and insulin all significantly decreased in the T2D group, while fasting ABAp significantly increased (Table 4). In this group, there was a negative correlation between FPG and ABAp (*r* = -0.683, *p =* 0.042), indicating that higher ABAp levels were observed in those subjects with lower FPG. Interestingly, fasting ABAp increased also in obese, but not diabetic subjects following BPD and this change was associated with a significant decrease of FPG, without a significant change in insulin (Table 4). Indeed, the ratio between post- and pre-BPD FPG values showed a significant (p = 0.044) moderate negative correlation (r = -0.614) with the ratio between post- and pre-BPD ABA values.

**Table 4:** Increased basal ABAp and decreased fasting glycemia in obese NGT and T2D subjects after BPD.

	NGT		T2D		
***N***	11		9		
**Males (%)**	4 (36)		5 (56)		
**Age (years)**	42.6±11.4		51.0±8.9		
	*Before BPD*	*After BPD*	*Before BPD*	*After BPD*	*p*
**BMI (kg/m** ^**2**^)	45.2±2.7	40.5±3.0	43.5±3.2	37.1±3.7	0.261*
	*p* = **0.004**		*p*<**0.001**		**0.047** #
**FPG (mg/dL)**	91.8±3.6	84.6±5.4	196.2±66.6	111.6±21.6	**0.003** *
	*p* = 0.064		*p* = **0.027**		**0.009** #
**Insulin (pmol/L)**	81.5±20.9	62.2±27.2	120.0±41.2	43.8±23.1	0.639*
	*p* = 0.269		*p* = **0.008**		0.062#
**HOMA-IR**	3.1±0.8	2.2±1.0	9.6±5.2	2.1±1.3	**0.030** *
	*p* = 0.150		*p* = **0.007**		0.467#
**ABAp (nmol/L)**	0.21±0.11	0.87±0.60	0.35±0.18	1.44±1.34	**0.045** *
	*p* = **0.028**		*p* = **0.037**		0.322#

The indicated parameters were assessed retrospectively on frozen plasma samples taken before and one month after bariatric surgery in subjects with NGT or with T2D. ABAp was measured by ELISA. BMI, body mass index; HOMA-IR, homeostasis model assessment of insulin resistance.

*unpaired t test NGT before BPD vs T2D before BPD

#unpaired t test NGT after BPD vs T2D after BPD.

Fasting ABAp values measured in obese NGT and T2D subjects (Table 4) were lower than those in NGT and T2D subjects of normal weight (Table 2); this discrepancy was not due to the different type of assay used to measure ABAp (ELISA and HPLC-MS in Table 4 and Table 2, respectively), since the two assays yielded similar results when performed on the same sample (not shown).

## Discussion

The main finding of the prospective part of this study is that the increase in ABAp that normally occurs after an oral glucose load is impaired both in patients with T2D and in women with GDM. In the latter ones, normalization of glucose tolerance after childbirth is paralleled by restoration of the ABAp response to oral glucose. Thus, impairment of the response of ABAp to hyperglycemia appears to be a common feature in T2D and GDM subjects. This finding points to a critical role for plasma ABA in the maintenance of a normal glucose tolerance and suggests new possible ABA-centered pathogenetic mechanisms that may underlie the diabetic condition. In principle, the failure of ABAp to increase following hyperglycemia in T2D and GDM may result from insufficient ABA synthesis or from dysfunctional regulation of ABA release in response to glucose. Regardless of the underlying mechanism, it is tempting to speculate that the loss of ABAp response to oral glucose contributes to the pathogenesis of T2D and GDM.

Concerning the tissue source of plasmatic ABA, pancreatic β-cells and adipocytes are both capable of releasing ABA upon stimulation with glucose *in vitro* [7], and the higher cell mass of the adipose tissue might make it an important determinant to fasting ABAp, particularly in obese subjects. Interestingly, the limited (approximately 10 and 15% in NGT and T2D subjects, respectively) reduction of body weight that occurred in both NGT and T2D subjects within the first month after BPD was associated with an increase, not a decrease, of ABAp (Table 4). This observation suggests an inhibitory effect on ABA secretion of one or more adipokines released from adipose tissue that is most readily lost after BPD.

BPD causes a substantial reduction in nutrient absorption in the small intestine [13]. Therefore, an alternative possibility is that stimulation of enteroendocrine cells by excess non-absorbed nutrients may result in an increased release of GLP-1 [14, 15], which in turn can stimulate ABA secretion from β-pancreatic cells [7]. Both hypotheses open new intriguing areas of investigation.

The higher mean value and wider distribution of the fasting ABAp in the T2D subjects compared to the NGT controls (Table 2) do not appear to be correlated with age or BMI in the T2D group and may reflect a heterogeneity of ABA-related dysfunctional mechanisms occurring in T2D, such as resistance to the glycemia-lowering effect of ABA (causing higher-than-normal basal ABAp levels), or impairment of the molecular mechanisms regulating the increase of ABAp in response to hyperglycemia (causing ABAp levels to be in the normal range despite hyperglycemia). At variance with what observed in T2D subjects, the fasting ABAp of the GDM subjects was lower compared to the NGT controls: this observation may suggest insufficient ABA release in response to hyperglycemia as the common pathogenetic mechanism underlying ABA dysfunction in GDM.

The prepartum basal ABAp in the pregnant NGT women (1.14±0.69 nM, n = 12) was not significantly higher (*p* = 0.054) than that measured in the male NGT subjects of Table 2 (0.75±0.54 nM, n = 30), although increasing the number of subjects may eventually yield a statistical significance; conversely, 8–12 weeks after childbirth, when glucose tolerance was restored to normal in the GDM subjects (all women were lactating), the postpartum basal ABAp of all the women tested was significantly higher than that of the NGT subjects (2.36±1.18 nM, n = 13; p = 2.45E-07). This observation, together with the fact that ABA is present in human milk (SB, unpublished observation), might suggest that a physiological increase of the ABAp occurs during lactation.

To gain further insight into ABAp deregulation in T2D, we also retrospectively measured fasting ABAp in obese subjects before and one month after BPD. This type of bariatric surgery is known to improve glucose tolerance, not only by reducing body weight and thus insulin resistance, but also by improving insulin secretion in response to glucose. Remarkably, improvement of glucose tolerance already occurs within one month after surgery [10, 16]. In our group of obese T2D subjects, BPD was followed by a significant decrease of both FPG and insulin, with a reduction of the HOMA-IR to normal values; this trend toward a normalization of glucose tolerance was paralleled by a significant increase of ABAp (Table 4). Thus, it can be postulated that the increase in the circulating levels of ABA may be one of the factors leading to improved glycemic control in T2D patients undergoing BPD. This hypothesis is supported by the fact that in NGT obese subjects too, BPD was followed by a significant increase in ABAp and a significant decrease in FPG, without significant modifications of insulin concentration and HOMA-IR (Table 4). As ABA stimulates GLUT-4 expression and its membrane translocation in adipocytes and myocytes *in vitro* [7], the beneficial effect of elevated ABAp on glycemic control might be secondary to increased peripheral glucose uptake.

In conclusion, this study provides evidence that the increase of ABAp normally observed in response to glucose is impaired in T2D and in GDM. In the latter form of diabetes, restoration of normal glucose tolerance after childbirth is accompanied by a normalization of the ABAp increase in response to glucose.

The demonstration that plasma ABA is altered/abnormal in T2D and GDM suggests a role for the dysregulation of ABAp response to hyperglycemia in the pathophysiology of these conditions and warrants further studies to test the new mechanistic hypotheses arising from this study.
